# From improvised to intentional: re-imagining the physician-scientist career path

**DOI:** 10.1172/jci.insight.204230

**Published:** 2024-04-13

**Authors:** Christopher S. Williams, Emily J. Gallagher, Daniel P. Cook, David Mankoff, Rebecca M. Baron, Christopher Pittenger, Jatin M. Vyas, Don C. Rockey, Patrick J. Hu, Ashley L. Steed, W. Kimryn Rathmell, Jeffrey R. Balser, Nancy J. Brown, John M. Carethers, Jonathan A. Epstein, Keith A. Choate, Peter J. Gruber, Tiffany C. Scharschmidt, ASCI Research Pathways Working Group, Kyu Y. Rhee

**Affiliations:** 1 Department of Medicine, Vanderbilt University Medical Center, Veterans Administration Health System, Vanderbilt Ingram Cancer Center, Nashville, Tennessee, USA.; 2 Department of Medicine, Icahn School of Medicine at Mount Sinai, New York, New York, USA.; 3 Department of Medicine, University of Iowa, Iowa City, Iowa, USA.; 4 Department of Radiology and Abramson Cancer Center, University of Pennsylvania, Philadelphia, Pennsylvania, USA.; 5 Division of Pulmonary and Critical Care Medicine, Brigham and Women’s Hospital and Harvard Medical School, Boston, Massachusetts, USA.; 6 Departments of Psychiatry, Neuroscience, and Psychology, Child Study Center, Center for Brain and Mind Health, and Wu-Tsai Institute, Yale University, New Haven, Connecticut, USA.; 7 Division of Infectious Diseases, Department of Medicine, Columbia University Vagelos College of Physicians and Surgeons, New York, New York, USA.; 8 Digestive Disease Research Center, Medical University of South Carolina, Charleston, South Carolina, USA.; 9 Department of Medicine, University of Colorado School of Medicine, Aurora, Colorado, USA.; 10 Department of Pediatrics, Washington University, St. Louis, Missouri, USA.; 11 James Cancer Hospital, The Ohio State University, Columbus, Ohio, USA.; 12 Department of Anesthesiology, Department of Medicine, Vanderbilt University Medical Center, Nashville, Tennessee, USA.; 13 Department of Internal Medicine, Yale School of Medicine, New Haven, Connecticut, USA.; 14 Department of Medicine, Moores Cancer Center, and Herbert Wertheim School of Public Health and Longevity Science, UCSD, San Diego, California, USA.; 15 Department of Medicine, Perelman School of Medicine at the University of Pennsylvania, Philadelphia, Pennsylvania, USA.; 16 Departments of Dermatology, Genetics, and Pathology, Yale School of Medicine, New Haven, Connecticut, USA.; 17 Department of Surgery, Yale School of Medicine, New Haven, Connecticut, USA.; 18 Department of Dermatology, UCSF, San Francisco, California, USA.; 19 See Supplemental Acknowledgments for details on the ASCI Research Pathways Working Group.; 20 Department of Medicine, Weill Cornell Medicine, New York, New York, USA.

## Abstract

The physician-scientist career has historically progressed through individual persistence and improvisation, as physician-scientists have navigated the demands of clinical practice combined with biomedical research without a clearly structured path. While this approach has sustained the field for several decades, individual determination is increasingly insufficient in the current climate, given the growing complexity within both clinical and research training, as well as potential disruptions to research funding and health care reimbursement. The 2025 American Society for Clinical Investigation/Alliance for Academic Internal Medicine/Burroughs Wellcome Fund Physician-Scientist Pathways Workshop convened national leaders and faculty at all career stages to assess existing structures and envision new and more deliberate approaches. Discussions highlighted the impact of NIH initiatives in supporting early careers, institutional vulnerabilities, and need for intentional investments in physician-scientist careers. Breakout sessions emphasized the importance of dedicated funding for physician-scientist pathways, mentorship, social supports, and national benchmarks for compensation and promotion for this unique career pathway. The physician-scientist career path now stands at a crossroads. Going forward, sustained investment, longer and more flexible funding mechanisms such as the R37 and R35 Maximizing Investigators’ Research Award programs, and transparent standards are required. Federal funding alone cannot ensure the stability of a physician-scientist’s career; therefore, new approaches and commitments from academic health centers, philanthropy, and industry will be essential to ensure the viability of this career. With coordinated, intentional strategic planning, the physician-scientist workforce can thrive and remain a driver of America’s biomedical research future.

## Abstract 2

The physician-scientist career is at a crossroads. The 2025 ASCI/AAIM/BWF Workshop called for intentional investment, flexible funding, mentorship, and institutional commitment to sustain this vital workforce.

## Graphical abstract

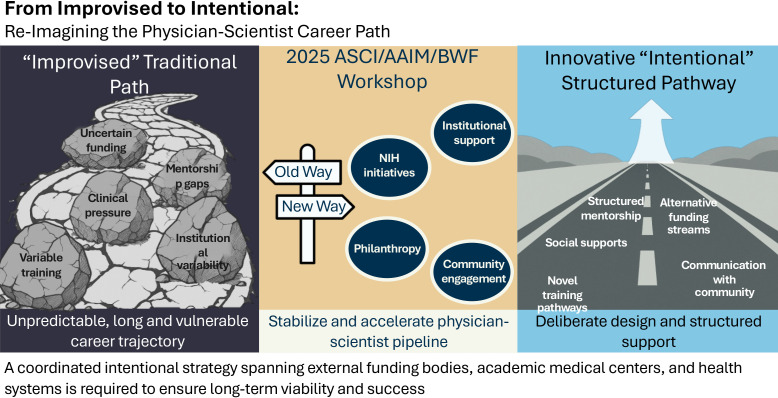

## Introduction

The career path of many physician-scientists has rested on the simultaneous pursuit of dual careers in clinical medicine and biomedical research (1, 2). Born in part from the diverse range of interests and approaches pursued by physician-scientists, this approach has led to career paths that, in many cases, are more a product of situational resourcefulness than strategic planning. This ad hoc approach has been further complicated by the growing complexity of each component career (3, 4). Recent and proposed disruptions to both federal research funding and health care reimbursement thus necessitate a paradigm shift away from situational adaptation and towards long-term intentional strategic planning.

The 2025 American Society for Clinical Investigation/Alliance for Academic Internal Medicine/Burroughs Wellcome Fund (ASCI/AAIM/BWF) Physician-Scientist Pathways Workshop was convened to respond to the fragmented and incompletely developed structure of the physician-scientist career path (5). The workshop sought to lay the foundation needed to develop a more structured career path for physician-scientists that better aligns the goals, resources, incentives, and expectations of institutions with those of individual physician-scientists across the career path. To do so, the workshop specifically took stock of the past 20 years of experience of NIH-funded physician-scientist training programs, surveyed the current and projected institutional landscape of potential strategies to support physician-scientists, and identified key challenges and opportunities on the topic of the physician-scientist career path from the perspective of workshop attendees. Workshop participants consisted of 106 stakeholders from more than 50 different academic institutions across the United States (plus one each from Canada and Korea), spanning residents, clinical fellows, MD-PhD, DO-PhD, research-in-residency, and Physician-Scientist Training Program (PSTP) directors, division chiefs, department chairs, executive vice presidents, deans, and CEOs; and clinical specialties ranging from anesthesiology to dermatology, internal medicine (and associated subspecialties), medical genetics, pathology, pediatrics, psychiatry, radiology, and surgery. Here, we highlight key takeaways from each.

## NIH initiatives supporting the physician-scientist workforce

Former National Cancer Institute (NCI) director and current CEO of the Arthur G. James Cancer Hospital and director of the Ohio State University Comprehensive Cancer Center, Kimryn Rathmell, first presented a retrospective analysis of NIH-funded physician-scientist training initiatives that highlighted the following key themes:

### Accelerating and stabilizing the career path of early-stage investigators is strategically critical.

A confluence of external factors (such as age at terminal degree, variable time spent in postgraduate clinical and scientific training, competing personal and financial needs and priorities) have contributed to a steadily increasing timeline towards independent R01-level funding (1). A lack of dedicated career-specific administrative and financial support mechanisms has further created systemic structural barriers that impact real and perceived readiness for scientific independence and cloud expectations associated with career development, further eroding the viability of this career path. Mechanisms to overcome these barriers have thus emerged as critical areas of unmet need to sustain a pipeline of future physician-scientists (6).

### NIH initiatives dedicated to physician-scientist career development have been, and continue to be, unequivocally successful.

Compared with the aggregate retention rate of postdoctoral T32 programs, the National Institute of General Medical Sciences–supported (NIGMS-supported) Medical Scientist Training Program (MSTP) T32 program has yielded a retention rate in an academic career path of approximately 65% (7). Investment in K01/K08/K23 programs has further yielded high rates of R01 applications (77%) and awards (50%), as exemplified by National Heart, Lung, and Blood Institute (NHLBI) K awardees from fiscal year 10 (FY10) to FY15 (8). Put in context, these rates closely rival those of NCI K99/R00 awardees (60%) (9).

K awards come with restrictions on salary support and required effort that can discourage applicants; mitigating these restrictions may enhance the physician-scientist pipeline. Aligning salary support to match committed research effort up to the legislative federal salary cap at the NCI in FY18 additionally tripled the number of K08 applications between FY17 and FY24, while a reduction in required committed effort for surgeon-scientists and other procedure-intensive scientists to 50% in FY20 increased the number of K08 applications at the NCI.

The NCI Continuing Umbrella of Research Experiences (CURE) K award program (1997–2021) demonstrated that the impact of K awards could be further enhanced by dedicated mentorship and preparation for independence, as reported by decreasing time to first R01 application and award (8).

A recently planned K program that provides up to 3 years of funding prior to a traditional career development award, the K32 New Academic Career Excellence (ACE) award, will test the impact of an earlier-stage K program.

### Shortening the time to first R01 for a physician-scientist may require extended and/or more flexible funding.

A key unanswered question is whether age at first awarded R01 and continued R01 funding are the best or only metrics to benchmark success of a training pathway. While higher paylines for early-stage investigators promote higher award rates, they do not stimulate more numerous applications. Higher award rates for first R01s similarly do not promote higher rates of success in achieving second R01s. In contrast, establishment of the R37 (MERIT Award) program for early-stage investigators by the NCI, which provided an additional 2 years of support beyond a typical 5-year R01, led to a nearly 40% increase in successfully achieving second R01 awards, corresponding to a nearly 35% absolute second R01 award rate by year 4. The establishment of the R35 Maximizing Investigators Research Award (MIRA) program at the NIGMS similarly provided investigators with additional time by extending R01 equivalent funding from 4 to 5 years and provided scientific flexibility that did not bind them to predefined specific aims. This program resulted in a lower age (1–2 years) at first independent award and is associated with a R01 renewal rate of nearly 80%. However, these programs have significant limitations; they are only supported by 6 NIH institutes (R35: NIGMS, NCI, NHLBI, and National Institute of Neurological Disorders and Stroke [NINDS]; R37: National Institute on Aging, National Institute of Allergy and Infectious Diseases, NINDS, and NCI). The R37 is not directly applied for but rather awarded through nomination by NIH program officers/staff for highly scored R01 applications. Only the NCI applies the R37 to Early Stage Investigators. The award structure involves a 5-year award with the potential for a 2-year extension at the budget of the final year of the initial period, with no specific funding cap or required level of effort commitment. The R35 requires a minimum effort commitment of 51% (depending on the institute), carries a maximum budget of $275K in direct costs, and is intended to support an investigator’s overall research program within a given institute, which limits the ability to obtain additional funding from the same institute. While the R35 can be renewed, resubmissions are not permitted, and the transition to R01 funding can create a potential funding cliff due to effort gaps. Establishment of similar additional mechanisms for physician-scientists thus represents a potentially fruitful but unrealized opportunity.

## Role of schools of medicine and academic health centers in sustaining physician-scientist careers

A panel discussion among nationally recognized deans/CEOs/executive vice presidents from 4 major US medical schools highlighted the following institution-level perspectives relevant to new potential models to support physician-scientist trainees and faculty.

Financial contributions from clinical revenue approaches half of what is provided by the NIH, making these institutions the second largest funder of biomedical research nationwide. At the same time, most academic health centers (AHCs) rely heavily on health care revenue to sustain clinical operations while cross-subsidizing the academic missions of teaching and research. As such, they are exquisitely vulnerable to disruptions of federal funds supporting either clinical care or research. Given the rising cost of health care and the significant federal budget challenges, revenue per unit of clinical service is declining, making it increasingly challenging and difficult to subsidize research and education. As academic medicine works to identify the means to buffer against federal clinical or research revenue challenges by identifying, developing, and incentivizing new external funding strategies, it is also imperative that AHC leaders and faculty work together to improve the overall cost efficiency of the research enterprise. The latter requires an array of efforts, ranging from more stringent effort allocation, spending, and purchasing policies, to improved cost effectiveness in program staffing and space allocations.

Recognizing that philanthropies may contribute at most 10% of the funding that has historically been provided by NIH, it was suggested that academic medicine will need to develop new models to incentivize other sources of support such as pharmaceutical company and other industry-based funding. In this regard, it was noted that private non-profit foundations often provide more funding opportunities for early-career investigators, but most do not provide significant facility and administration costs. It was similarly noted that AHCs may need to develop more centralized policies and fund-flow models capable of directing funds with more flexibility, including endowments in strategic priority areas.

Among research-intensive schools of medicine and AHCs, physician-scientists were identified as among the most unique and valuable assets. As such, there was universal support for prioritization of junior faculty physician-scientists, although it was also recognized that schools of medicine may need to develop new training paradigms and pathways to sustain the pipeline of early-stage physician-scientists that not only improve success rates, but also streamline training to reduce its overall duration and cost. The need for better public communication about the importance of research and specific role of AHCs in advancing clinical medicine was finally discussed with an emphasis on the unique, essential role the physician-scientist plays currently and for the future.

## Identifying structural needs to strengthen the physician-scientist pathway

Breakout sessions consisting of participants from all faculty career stages, broadly representing multiple US geographic regions and a range of medical fields, provided focused perspectives on the following topics:

### Dedicated institutional funding commitments and programs for physician-scientist careers.

Several schools of medicine have created — and more should create — institutional physician-scientist umbrella programs that are broadly inclusive of all physicians doing investigative work (and include lecture series, work-in-progress talks, named societies that support the physician-scientist environment, websites), even if they are low-cost/cost-neutral, due to their ability to create a critical mass of investigators that can foster scientific and career development synergies and a professional community within and across career stages. Isolation from research during residency training, in particular, can contribute to attrition from investigative careers. Preventing this isolation may require implementing dedicated mechanisms, including but not limited to R38-type programs (Stimulating Access to Research in Residency; StARR). Moreover, deliberate integration and partnership of physician-scientists with clinical educators can help to sustain academic curiosity and scientific interest among trainees. This is a low-cost, high-value investment that promotes exposure to investigative perspectives throughout clinical training, thus cultivating an investigative mindset that may also facilitate recruitment of “late-bloomers” into physician-scientist careers. Funding to support physician-scientists will nonetheless almost certainly be essential and will need to come from philanthropy, novel clinical fund-flow models, and innovative partnerships with pharmaceutical companies and/or the biotechnology industry, in addition to governmental sources. Such programs should be intentional and measurably impactful and should be linked to national benchmarks, including around physician-scientist compensation, and professional expectations.

### Bolstering mentorship and research training pathways will clearly help support the physician-scientist endeavor.

Developing critical masses of mentors and trainees — at both the institutional and national level — is essential but will likely require creating a balance between intentionality and incentivization of mentor engagement as well as mechanisms capable of accommodating different entry points into the career pathway. As of now, the majority of mentors provide uncompensated support for trainees and therefore the system depends on the ability of these faculty members to take time away from their own stressful career support to do this critically important work. Creative mechanisms for direct support of mentoring, including but not limited to dedicated endowments, are thus equally critical. The need for such a balance is particularly vital for trainees during and across the instructor/lecturer period of career development.

### Financial challenges in the transition to independence of early-career physician-scientists.

Trainees typically have little to no knowledge of or access to the financial, managerial, and administrative resources or contractual terms needed to successfully transition to research independence. As such, they are often dependent on anecdotal or second-hand experiences and/or an empirical “limit testing”–based approach. Salary expectations and appropriate benchmarks/standards of parity are particularly unclear while existing family support resources are often insufficient and/or incompatible with clinical/scientific obligations, highlighting a need for more structure and transparency.

## Concluding remarks and perspectives from the workshop

Reflecting on the foregoing discussions, this year’s workshop not only highlighted many of the key challenges facing the physician-scientist career path and the progress that has been made toward overcoming them, but also identified new challenges that represent topics of focused discussion for future workshops (Table 1). Stated differently, the physician-scientist career path currently stands at a crossroads. The way forward requires deliberate design, sustained investment, and coordinated action across multiple stakeholders. Resources must focus on career development awards that have demonstrated success in supporting physician-scientists to the next phase of their careers. The physician-scientist pathway requires extended funding periods and greater flexibility, making programs like R35 MIRA and R37 models the norm rather than exceptions. National benchmarks for compensation, effort expectations, and career progression must be established to provide transparency for both individuals navigating physician-scientist career paths and the institutions that support them. Finally, recognizing that federal funding alone cannot sustain the physician-scientist workforce, institutions must actively prioritize physician-scientist support and cultivate alternative funding streams through philanthropy, industry partnerships, and clinical innovation to ensure long-term viability (10).

The transformation required is not merely operational but also cultural. It demands evolving from viewing physician-scientists as talented individuals juggling research, patient care, administration, and education to recognizing them as professionals requiring distinct training pathways, support structures, and success metrics (1, 2, 6). In this regard, it is particularly notable that, despite inherent differences in their perspectives, a former NIH institute director, 4 deans/vice presidents, and a participant panel spanning all stages of career development independently identified support during the transition from trainee to independent investigator as among the most pressing, yet unrecognized, areas of unmet need. Achieving such transformation requires coordinated action and collaboration among federal funding agencies, schools of medicine, training programs, and individual physician-scientists. The evidence presented at the 2025 ASCI/AAIM/BWF Physician-Scientist Pathways Workshop suggests that with strategic reforms — particularly those that provide extended funding, greater flexibility, and defined career pathways — the physician-scientist workforce can not only survive but thrive into the future.

Many schools of medicine and academic health centers have made focused investments to support the physician-scientist pipeline and to shorten the timeline to independence. All have been navigating a challenging policy environment over the last year, marked by budgetary volatility, regulatory changes, and campus climate concerns. In response, many centers have taken targeted measures to stabilize the physician-scientist pipeline. We present efforts underway at many AHCs prior to policy changes, and responses implemented after.

## Conflict of interest

JRB reports serving on the Board of Directors at CVS Health. NJB reports ownership in Alnylam and Sabra Healthcare Reit and spouse’s ownership in 3M Corporation, Abbvie, Albermarle, Becton Dicksonson, Brentwood Capitol Advisors, Eli Lilly, Healthcare Reality Trust, Johnson & Johnson, Kenvue Inc., NCN Capital, National Health Advisors, Walgreens Boots Alliance, and Waters. NJB reports income from Alnylam Pharmaceuticals.

## Funding support

This work is the result of NIH funding, in whole or in part, and is subject to the NIH Public Access Policy. Through acceptance of this federal funding, the NIH has been given a right to make the work publicly available in PubMed Central.

- CSW notes support from NIH MSTP grant T32GM152284, Vanderbilt StARR (NIH grant T32GM152284), Vanderbilt Ingram Cancer Center (NIH grant P30CA068485), and a VUMC Directorship.
- EJG reports support from the Mount Sinai StARR programs (NIH grants R38HL172261 and R38AI181012).
- DPC notes support from NIH career training grant K08AI181763.
- CP acknowledges support from NIH grants T32MH019961, R25MH0715834, K24MH121571, and from the state of Connecticut through its support of the Ribicoff Research Facilities at the Connecticut Mental Health Center.
- DAM notes support from NIH grant T32EB004311, UPenn Research Track Radiology Residency, and NIH grant P30CA016520 (Abramson Cancer Center Support Grant).
- JMV reports support from NIH grant R38AG070229.
- ALS acknowledges support from the Burroughs Wellcome Fund.
- TCS notes support to the UCSF Dermatology PSTP from NIH grant T32AR007175.
- DCR reports support from NIH grants UE5DK137316 and R25DK137316.
- NJB acknowledges support from NIH grant R01HL145293.
- KYR acknowledges support from the Burroughs Wellcome Fund Physician-Scientist Institutional Award to Weill Cornell Medicine Physician-Scientist Academy and Tri-I StARR (NIH grant R38AI174255).

## Supplementary Material

Supplemental data

## Figures and Tables

**Table 1:**
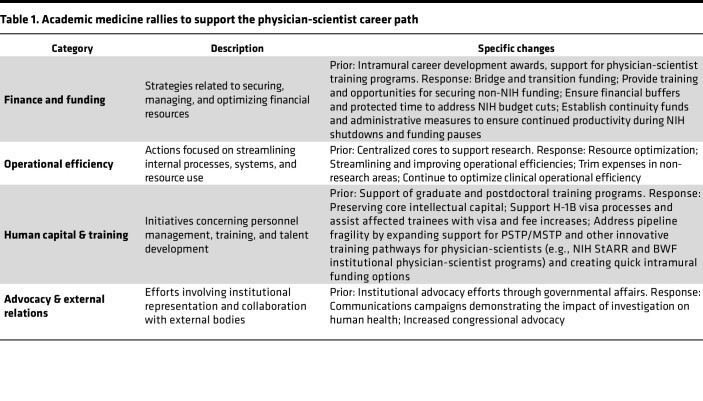
Academic medicine rallies to support the physician-scientist career path

## Version 1 04/14/2026

In-Press Preview

## Version 2 06/08/2026

Electronic publication
