# Supplementary material for: From improvised to intentional: re-imagining the physician-scientist career path
Source: JCI Insight. 2024 Apr 13;11(11):e204230. doi: 10.1172/jci.insight.204230 (PMC13313553; doi:10.1172/jci.insight.204230)
Supplement: Supplemental data [file jciinsight-11-204230-s277.pdf]

Supplemental Acknowledgments

ASCI/AAIM/BWF Physician-Scientist Research Pathways Working Group Members:

- Christopher S. Williams, Department of Medicine, Vanderbilt University Medical Center, Veterans Administration Health System, Vanderbilt Ingram Cancer Center, Nashville, Tennessee, USA.
- Emily J. Gallagher, Department of Medicine, Icahn School of Medicine at Mount Sinai, New York, New York, USA.
- Daniel P. Cook, Department of Medicine, University of Iowa, Iowa City, Iowa, USA.
- David Mankoff, Department of Radiology and Abramson Cancer Center, University of Pennsylvania, Philadelphia, Pennsylvania, USA
- Rebecca M. Baron, Division of Pulmonary and Critical Care Medicine, Brigham and Women's Hospital and Harvard Medical School, Boston, Massachusetts, USA
- Christopher Pittenger, Departments of Psychiatry, Neuroscience, and Psychology, Child Study Center, Center for Brain and Mind Health, and Wu-Tsai Institute, Yale University, New Haven, Connecticut, USA
- Jatin M. Vyas, Division of Infectious Diseases, Department of Medicine, Columbia University Vagelos College of Physicians and Surgeons, New York, New York, USA
- Don C. Rockey, Digestive Disease Research Center, Medical University of South Carolina, Charleston, South Carolina, USA.
- Patrick J. Hu, Department of Medicine, University of Colorado School of Medicine, Aurora, Colorado, USA.
- Ashley L. Steed, Department of Pediatrics, Washington University, St. Louis, Missouri, USA.
- W. Kimryn Rathmell, James Cancer Hospital, The Ohio State University, Columbus, Ohio, USA
- Jeffrey R. Balser, Vanderbilt University, Nashville, Tennessee, USA
- Nancy J. Brown, Department of Internal Medicine, Yale School of Medicine, New Haven, Connecticut, USA
- John M. Carethers, Department of Medicine, Moores Cancer Center, and Herbert Wertheim School of Public Health and Longevity Science, UCSD, San Diego, California, USA.

- Jonathan A. Epstein, Department of Medicine, Perelman School of Medicine at the University of Pennsylvania, Philadelphia, Pennsylvania, USA.
- Keith A. Choate, Departments of Dermatology, Genetics, and Pathology, Yale School of Medicine, New Haven, Connecticut, USA
- Peter J. Gruber, Department of Surgery, Yale School of Medicine, New Haven, Connecticut, USA.
- Tiffany C. Scharschmidt, Department of Dermatology, UCSF, San Francisco, California, USA
- Kyu Y. Rhee, Department of Medicine, Weill Cornell Medicine, New York, New York, USA.
- Olujimi Ajijola, MD, PhD, Department of Medicine, University of California, Los Angeles, Los Angeles, California, USA.
- Paige Cooper Byas, PhD, Burroughs Wellcome Fund
- Holger K. Eltzschig, MD, PhD, Department of Anesthesiology, McGovern Medical School, University of Texas
- Charles W. Emala, MS, MD, Department of Anesthesiology, Columbia University
